# Murine hepatoblast-derived liver tumors resembling human combined hepatocellular-cholangiocarcinoma with stem cell features

**DOI:** 10.1186/s13578-020-00395-2

**Published:** 2020-03-13

**Authors:** Xiong Cai, Heli Li, David E. Kaplan

**Affiliations:** 1 Division of Gastroenterology and Hepatology, Department of Medicine, University of Pennsylvania, 3400 Civic Center Drive, PCAM GI 7S, Philadelphia, PA 19104-6145 USA; 2 Department of Hepatobiliary Surgery, Union Hospital, Tongji Medical College, Huazhong University of Science and Technology, 1277 Jiefang Ave., Wuhan, 430022 China; 3 Department of Immunology, Tongji Medical College, Huazhong University of Science and Technology, Wuhan, 430030 China

**Keywords:** Mouse, Human, Cholangiohepatoma, Progenitor, Heterogeneity, p53

## Abstract

**Background:**

Combined hepatocellular-cholangiocarcinoma (CHC) is a primary hepatic malignancy with heterogeneously combined histological features of putative hepatic progenitor cells (HPC) origin. We describe a mouse model that exhibits the heterogenous histological and phenotypic finding similar to human CHC.

**Methods:**

We injected hepatoblasts isolated from p53^−/−^ C57BL/6 mice into syngeneic wild-type pre-conditioned C57BL/6 mice. We confirmed that p53^−/−^ murine hepatoblasts act as tumor-initiating cells (TICs) that generate CHC both in situ and within metastases. For comparative pathological study, 8 human cases of CHC with stem cell features were recruited by immunohistochemistry and multicolor fluorescence immunostaining.

**Results:**

We identified corresponding areas in murine tumors matching each WHO criteria-described subtype of human CHC. In both murine and human tumors, HPC-like cells in tumor nests and associated stem cell features/traits are suggested histologically to be the progenitor origin of the cancer

**Conclusions:**

The pathological characteristics of murine tumors recapitulate human CHC with stem cell features. These data provide additional comparative pathological evidence that CHC with stem cell features originate from HPCs and validate a model to study this cancer type in vivo.

## Background

Combined hepatocellular-cholangiocarcinoma (CHC) represents fewer than 3% of human primary liver malignancies. CHC manifests combined histological features of both hepatocellular carcinoma (HCC) and cholangiocarcinoma (CC) [1]. Although this unique tumor was first described in 1903 [2], due to its rarity and remarkable heterogeneity, detailed criteria for diagnosis and subclassification continue to be refined. Currently, most pathologists regard only Type C tumors defined by Allen and Lisa [3] and/or Type II (transitional) tumor defined by Goodman [4] that exhibit a mixture of the hepatocellular and biliary differentiation as CHC.

Although the debate whether hepatocellular carcinoma (HCC) arises from stem cells that undergo malignant transformation or alternatively from de-differentiated, transformed mature hepatocytes continues [5], most evidence suggest that CHC specifically arises from hepatic progenitor cells (HPC) [6, 7]. The co-existence of hepatocellular and cholangiocellular components in CHC suggests origin from a common progenitor. Moreover, HPC-like tumor cells, identified as small, oval-shaped cells with hyperchromatic nuclei and scant cytoplasm, can frequently be found within CHC [8]. These HPC-like tumor cells are also characterized by co-expression of multiple progenitor/stem cell markers [9]. Nevertheless, the cross-sectional nature of the pathological studies precludes conclusive establishment of HPCs as tumor-initiating cells in human CHC.

During the last 2 decades, there has been active research on tumorigenic role of liver parenchymal cells in experimental animals. A milestone study demonstrated that HPCs, hepatoblasts, and mature hepatocytes all can be transformed in vivo [10], suggesting that primary liver malignancies might derived from multiple stages of hepatocyte differentiation. Lineage tracing technology has allowed researchers to further explore the cellular origin of hepatocarcinogenesis in multiple animal models [11, 12], however to date consensus has yet to be reached as to whether progenitor cells or de-differentiated hepatocytes are the primary tumor initiating cells (TICs) in hepatic malignancies. Variability of the results from various studies may due to diverse genetic background of animal models, heterogeneous approaches of inducing hepatic damage, and/or challenges in finding optimal biomarkers for lineage tracing [12].

A minority of these studies have observed tumors with the marked heterogeneity typical of CHC [13–17]. One recent study has indeed linked HPC origin to the heterogeneity of generated tumors and suggested that both the differentiation state of the TIC as well as specific genetic alterations could affect the phenotypic diversity of the resulting tumors [10]. It also has been suggested that chronic liver inflammation also contributes to tumor phenotypes [18].

Based upon these findings, we studied tumor heterogeneity that developed after seeding tumorigenic hepatic progenitor cells (isolated p53^−/−^ C57BL/6 fetal hepatoblasts) trans-splenically into livers of inflammatory pre-conditioned syngeneic C57BL/6 mice [14]. In this oncogene-driven mosaic model, we observed tumor nodules with CHC-like mixed histological features both within the liver and in metastatic sites. In the present paper, we present the side-by-side results of a comparative pathological study of these murine hepatoblasts-derived liver tumors and human CHC with stem cell features. The study focuses on heterogeneity of tumors and reveals correspondence in histological and phenotypic finding, aiming to closing the gap between the mouse model and human CHC.

## Materials and methods

### Ethics

All experiments with mice were performed under a protocol approved by Institutional Animal Care and Use Committee (IACUC) of the University of Pennsylvania, the Corporal Michael J. Crescenz VA Medical Center, and Second Military Medical University and received humane care according to the criteria outlined in the “Guide for the Care and Use of Laboratory Animals”.

### Generation of hepatoblasts

B6.129S2-*Trp53*^*tm1Tyj*^/J (p53^−/−^ or p53^wt/wt^) mice embryos were harvested at E13.5. Purification of E-Cadherin^+^ hepatoblasts from embryonic liver suspensions was performed using the MACS^®^ magnetic cell-sorting system (Miltenyi, Auburn, California) with the rat anti-mouse E-Cadherin (ECCD-1) antibody (Calbiochem, San Diego, California) as described [19]. Detailed description of cell purification, identification, culture, and retroviral infection can be found in Additional files 1, 2.

### Recipient mice

**Fig. 1:**
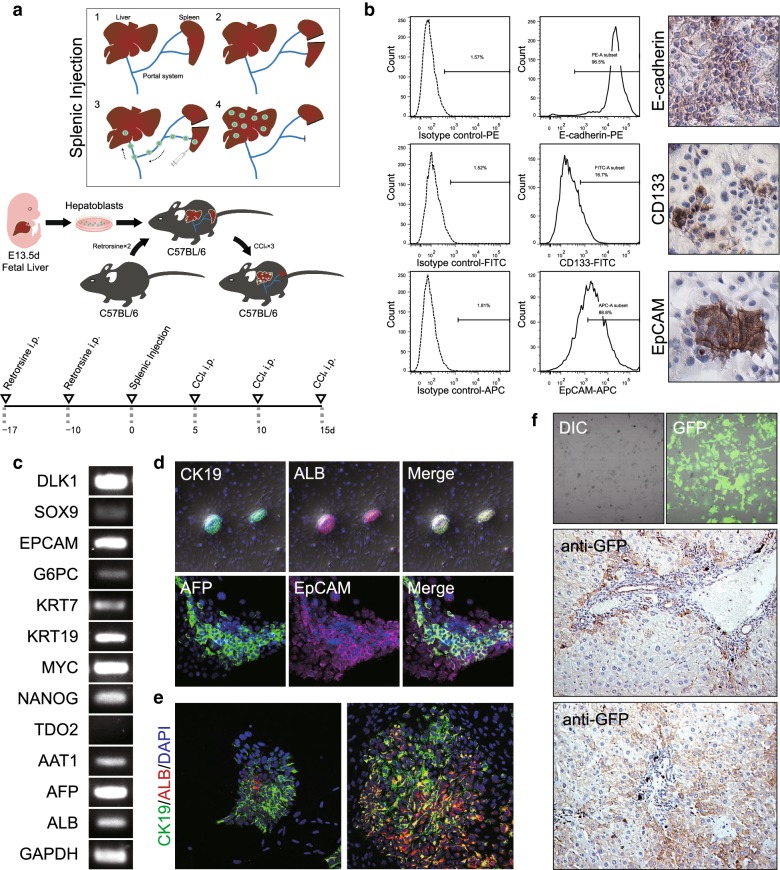
Characterization of E-cadherin^+^ fetal liver cells as bipotential progenitor cells. **a** The experimental schema for p53^−/−^ hepatoblast isolation, creation of hepatic inflammatory environment, and trans-splenic intrahepatic delivery of hepatoblasts for either liver repopulation or tumor formation. **b** Expression of hepatic progenitor cell-associated surface markers examined by flow cytometry after 5-day culture with immunohistochemistry confirmation. Histograms represent expression of E-cadherin (upper), CD133 (middle), and EpCAM (lower) relative to isotype controls. Cells cultured on Matrigel-coated were fixed for immunocytochemistry by a two-step indirect protocol for confocal microscopy. Representative images showing expression patterns of E-cadherin, CD133 and EpCAM are shown in the right panel. **c** Reverse-transcription PCR expression of hepatocytic (*AAT1*, *AFP*, *ALB*, and *TDO2*), cholangiocytic (*G6PC*, *KRT7*, and *KRT19*) and progenitor cell markers (*DLK1*, *SOX9*, *EPCAM*, *MYC*, and *NANOG*) by purified E-cadherin^+^ fetal liver cells after 5 days culture. **d** Immunofluorescence demonstrating that E-cadherin^+^ fetal liver cells express ALB/CK19 as well as AFP/EpCAM. Upper representative view shows two colonies derived from single E-cadherin^+^ fetal liver cells co-cultured with STO feeders. Lower representative view shows a colony derived from a single E-cadherin^+^ fetal liver cell cultured on Matrigel. **f** Single E-cadherin^+^ fetal liver cell-derived clones contained exclusively ALB-positive hepatocytes and CK19-positive cholangiocytes after a 7-day spontaneous differentiation after co-culture with STO feeders in basal media. Left image shows a colony manifesting mostly cholangiocellular differentiation. The right image showed bi-directional differentiation. **f** In vivo repopulation and differentiation of E-cadherin-positive fetal liver cells. The sections of the recipient liver were subjected to observation of GFP-immunoreactive cells forming bile duct structures (upper) and liver parenchyma (lower). Mice received splenic injection of HBSS as controls

Wild type female C57BL/6 mice (Jackson Laboratories, Rochester, New York), 8–12 weeks of age, weighing 20–30 g were used as recipients for intra-splenic injection. Retrorsine (70 mg/kg i.p.) was administered 17 days and 10 days prior surgery to inhibit hepatocyte proliferation, followed by CCl_4_ (0.5 mL/kg i.p.) administration for 3 times with 5 days interval to induce hepatic inflammation (Fig. 1a) per a previously described protocol [20]. A single-cell suspension hepatoblasts was injected into the lower splenic pole following surgical procedures described previously [21]. Ten mice were transplanted with non-retroviral transfected p53^−/−^ hepatoblasts for monitoring of tumor formation [21]. Ten control mice were transplanted with non-retroviral transfected p53^wt/wt^ hepatoblasts. Body weight, physical appearance, measurable clinical signs, unprovoked behavior and response to external stimuli were monitored daily after surgery. 10 weeks after surgery, all mice were sacrificed for pathological examination. One mouse died spontaneously of advanced liver tumor at week 10. Three additional mice received p53^−/−^ retrovirally GFP^+^-transduced hepatoblasts and were sacrificed at day 7 to measure donor cell repopulation and in vivo differentiation. Three mice that received transsplenic injection of HBSS served as negative controls.

### Patients and clinical data

From January 1997 to December 2003, 88 patients who underwent hepatectomy in the Department of Hepatic Surgery, Eastern Hepatobiliary Surgery Hospital, Second Military Medical University and who were postoperatively confirmed as CHC were reviewed elsewhere. After excluding patients with recurrent tumors or insufficient surgical specimen, 8 of remaining 80 patients diagnosed as CHC with stem-cell feature were recruited. Informed consent was obtained from each patient under a protocol approved by the Hospital Research Ethics Committees. Patient clinical characteristics are presented in Table 1.

**Table 1:** Clinical data of 8 patients diagnosed as combined hepatocellular-cholangiocarcinoma with stem cell feature

Case	Age at surgery	Sex	Etiology	Liver fibrosis (Ishak stage 0–6)	Serum AFP (µg/L)	Serum CA19-9 (µg/L)	Tumor location (segments)	Tumor number	Maxi tumor diameter (cm)	Diagnosis according to WHO criteria	Minor component within intermediated areas	Prognosis
1	50	M	HBV	6	51.2	27.2	IV, V	1	2	CHC with stem cell feature, typical type,	Intermediate-cell foci	Lost follow-up
2	64	M	HBV	2	7.6	N/A	VI, VII	1	10	CHC with stem cell feature, intermediate cell type	None	Recurrence was diagnosed by CT scan in liver at 11 mos. Patient died at 14 mos
3	49	M	HBV	6	> 1000.0	0	VIII	1	6.5	CHC with stem cell feature, intermediate cell type	Cholangiolocellular foci	Recurrence in liver was diagnosed by CT scan at 13 mos. Patient died soon after recurrence
4	52	M	HBV	3	61.2	0.1	V	1	2	CHC with stem cell feature, intermediate cell type	Foci with typical type appearance	None recurrence was found at 74 mos
5	39	M	HBV	6	> 1000.0	26.2	IV	1	4.5	CHC with stem cell feature, intermediate cell type	None	New tumor was diagnosed by CT and DSA in liver at 2 mos. patient received TACE, died at 5 mos
6	55	M	HBV	6	561.5	17.5	VI	1	5.3	CHC with stem cell feature, intermediate cell type	Foci with typical type appearance and cholangiolocellular foci	New tumor was diagnosed by CT at 42 mos. Patient then received repeat hepatectomy. Resected specimen was pathologically diagnosed as HCC. None recurrence was found at the time of follow-up closure
7	42	M	HCV	6	95.1	39.9	II, III, IV	3	9.2	CHC with stem cell feature, intermediate cell type	None	Patient died of hemorrhage with no evidence of recurrence at 4 mos
8	53	M	HBV	6	> 1000.0	31.8	II, III, IV	3	5.5	CHC with stem cell feature, intermediate cell type	None	New tumor was diagnosed by CT and DSA in liver at 2 mos. After receiving TACE for several times, patient died at 23 mos

*AFP* alpha-fetoprotein, *CA19-9* carbohydrate antigen 19-9, *CHC* combined hepatocellular-cholangiocarcinoma, *CLC* cholangiolocarcinoma, *CT* computed tomography, *HBV* hepatitis B virus, *HCV* hepatitis C virus, *DSA* digital subtraction angiography, *HCC* hepatocellular carcinoma, *TACE* transarterial chemoembolization, *WHO* World Health Organization

### Diagnostic criteria of CHC for both rodents and human

The definitive diagnosis of CHC can only be established by histopathology. The latest WHO classification (4th edition) defined in 2010 provides detailed subtypes and unified diagnostic criteria of human CHC [22]. Detailed diagnostic criteria and representative images have been described in previous publications [1, 23]. For rodents, criteria have been suggested by the International Agency for Research on Cancer (IARC) of WHO in 2001. In addition, a standardized guideline established by the International Harmonization of Nomenclature and Diagnostic Criteria for Lesions in Rats and Mice (INHAND) project provides valuable diagnostic features [24]. In the current study, histological diagnosis and nomenclature of the tumors comprehensively referred to the WHO criteria for both human and rodents.

## Results

### Characterization of cultured hepatoblasts

To determine whether cultured hepatoblasts retain expression profiles consistent with bi-potential liver progenitors, expression of hepatic progenitor cell-associated surface markers (e.g., EpCAM, CD133, E-cadherin) was examined by flow cytometry and confirmed by immunocytochemistry after 5-days in vitro (Fig. 1b). EpCAM expression was observed in the cell membrane of the majority of cells, and 88.8% of cells expressed EpCAM by flow cytometry. By contrast, only a minority (16.7%) expressed CD133. By RT-PCR after 5 days of culture, purified E-cadherin^+^ fetal hepatoblasts expressed Protein delta homolog 1 (*DLK1*), *SOX9*, *EPCAM*, Albumin (*ALB*), α1-antitrypsin (*AAT1*), glucose-6 phosphatase (*G6PD*), cytokeratin 19 (CK19, *KRT19*), cytokeratin 7 (CK7, *KRT7*), *MYC*, *NANOG*, and alphafetoprotein (*AFP*) but did not express the hepatocytic differentiation marker tryptophan 2,3-dioxygenase (*TDO2*) (Fig. 1c). Co-expression of these markers was confirmed by double-fluorescence immunostaining (Fig. 1d). Consistent with bipotentiality, p53^−/−^ hepatoblasts cultured on STO feeders displayed co-localization of the hepatocytic marker ALB and the biliary epithelial marker CK19, with > 90% being ALB and CK19-double positive cells. Further, we performed immunofluorescence on liver progenitor cells cultured on Matrigel surface using EpCAM and AFP monoclonal antibodies, co-expression of two antigens was also observed suggesting a hepatic TIC-like trait [9]. Spontaneous differentiation was then induced over 7-days by withdrawing growth factors, after which single E-cadherin^+^ fetal liver cell-derived clones contained either exclusively ALB-positive hepatocytes or CK19-positive cholangiocytes (Fig. 1e). As shown in Fig. 1f, when GFP-transduced hepatoblasts differentiated in vivo*,* GFP^+^ staining was observed in both morphologically mature hepatocytes and bile canalicular-like structures confirming the capacity of donor E-Cadherin^+^ hepatoblasts to re-populate recipient liver with both hepatocytic and cholangiocytic structures. Sections from HBSS controls did not show any GFP^+^ staining (data not shown). Collectively, the results of the expression profiles and differentiation assays strongly suggest that E-Cadherin^+^ fetal liver cells are indeed bipotential liver progenitor cells.

### Development of intrahepatic tumor and extrahepatic metastases in murine

**Fig. 2:**
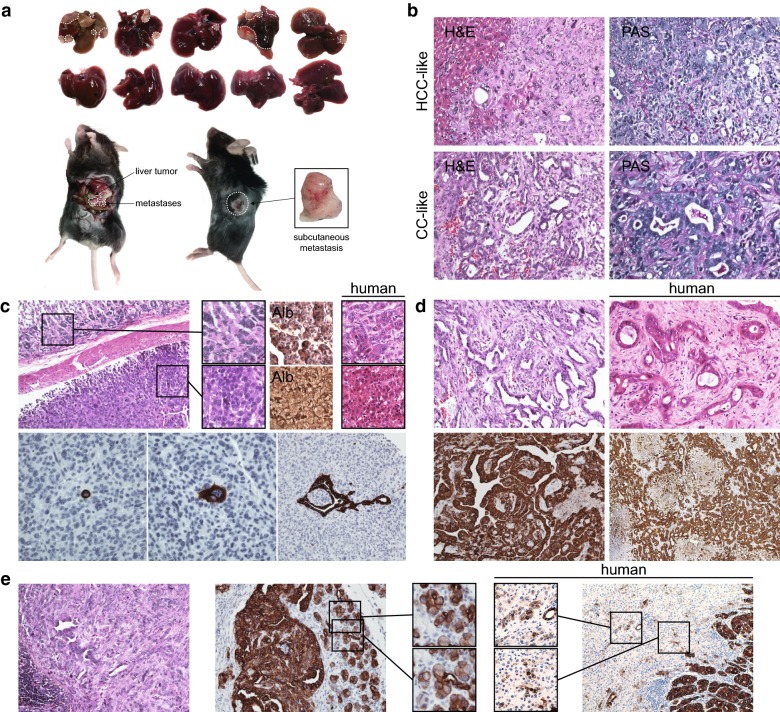
Tumor formation in situ and metastasis by p53^−/−^ hepatoblasts. **a** Images of livers explanted from wild-type CCl_4_-primed mice that receipted trans-splenic injection of p53^−/−^ hepatoblasts are shown in upper panel. Metastases in abdominal cavity and left back are also shown. Tumor regions are highlighted by dash lines. **b** Hematoxylin–eosin staining of areas of tumor show hepatocellular (HCC-like area, upper left) or cholangiocellular differentiation (CC-like area, lower left). Briefly, HCC-like cells were poorly differentiated with compact trabecular pattern. The tumor cells show a “pushing-border” invading into adjacent normal hepatic tissue. Representative HCC-like area without obvious mucus production is shown in upper right panel. Within CC-like area shown, the invasive adenocarcinoma was moderately differentiated with glandular growth pattern. Mucus production is highlighted by Periodic Acid-Schiff staining within irregular lumens in the lower right panel. Images were from mouse no. 266. **c** Hematoxylin–eosin staining of two metastases from the abdominal cavity separated by fibrous capsule shows heterogeneous histology (mouse no. 263). Higher magnifications of the boxes highlight the well differentiated neoplastic hepatocytes with clusters of small tumor cells around (upper) and moderately differentiated HCC (lower). The hepatic origin of the metastases is confirmed by albumin staining. Corresponding human tumor samples (patient no. 5) are shown for comparison. In the lower panels, focal small tumor cells, poorly differentiated tumor cells, and well-differentiated adenocarcinomas explanted from recipient mice are highlighted by CK19 staining. **d** Histologic profiles of subcutaneous metastasis are shown (mouse no. 266). Hematoxylin–eosin staining of the margin area of one metastasis shows ill-defined glandular structure embedded in broad desmoplastic stroma. In the center area of metastasis, well-moderately differentiated adenocarcinoma highlighted by CK19 staining is associated with less fibrous stroma. The corresponding histologic appearances of CC-like areas in human tumors are presented for comparison (patient no. 8). **e** Representative splenic tumors (mouse no. 266) mimic the multicentric occurrence features of human combined hepatocellular-cholangiocarcinoma (patient no. 8). Higher magnifications of the boxes show striking similarity to ductular reactions with hepatocyte regeneration in well differentiated HCC areas of CK19 staining sections from both human and mice

All mice that received trans-splenic hepatoblast injections uneventfully recovered. Ten weeks after cell transplantation, one mouse died of advanced liver tumor; at that point the other 9 mice were sacrificed for macroscopic and microscopic examination. As shown in Fig. 2a, 5 of 10 mice developed overt tumor nodule formation in situ*,* three with multinodular tumors and two with solitary lesions. One mouse with a solitary hepatic tumor was found to have developed a subcutaneous metastasis on left back as well as the upper pole of the spleen. Two of three mice with multinodular tumor in liver manifested near complete replacement of the liver by tumors. One of these mice also developed extensive abdominal cavity tumor metastases. No lung or bone metastases were observed either macro- or microscopically in any mouse (data not shown). No tumors were found in p53^wt/wt^ hepatoblast-transplanted control mice (data not shown).

### Histopathological findings of intrahepatic tumors in murine: consistent with CHC diagnosis

**Fig. 3:**
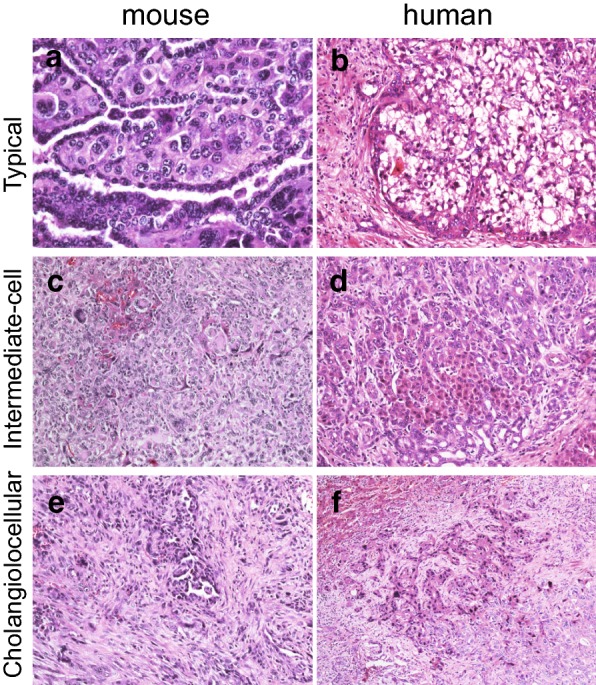
Comparison of p53^−/−^ murine and human primary liver neoplasms according to WHO criteria for human. Representative images of typical (**a** mouse no. 263), intermediated-cell (**c** mouse no. 271), and cholangiolocellular foci (**e** mouse no. 266) in murine tumors based on the WHO criteria descriptions are presented side by side with images of corresponding intermediate area from typical subtype (**b** patient no. 1), intermediate-cell subtype (**d** patient no. 5), and cholangiolocellular foci (**f** patient no. 3) of human combined hepatocellular-cholangiocarcinoma with stem cell features

Grossly, the intrahepatic tumors appeared macroscopically as well-defined, white, solid masses, measuring 2–12 mm (Fig. 2a). As illustrated in Figs. 2b and 3, H&E staining sections of the tumors showed extremely varied appearances, with several distinct growth patterns. The tumors were composed of following three elements: HCC-like areas, CC-like areas, and intermediate areas, consistent with CHC diagnosis. The HCC-like areas were poorly differentiated with a compact pattern. The malignant hepatocytes showed hyperchromatic nuclei and mitotic figures, and a “pushing-border” invaded into the adjacent normal hepatic tissue (Fig. 2b, upper panels). Trabecular architecture was absent in our observations. With respect to CC-like areas, the invasive adenocarcinoma was well-to-moderately differentiated with glandular, papillary and/or solid growth patterns (Fig. 2b, lower panels). Mucus production was identified within irregular lumens constructed by tall columnar neoplastic epithelial cells (Fig. 2b). Fibrous stroma formation was not marked. The majority of tumor areas could not be specifically characterized as HCC or CC, but displayed a basophilic cell population with hyperchromatic, oval nuclei consistent with intermediate areas.

### Histopathological findings of extrahepatic metastases in murine: variations in organ specificity

As shown in Fig. 2c–e, not only tumors in situ, but also the extrahepatic metastases showed a varied pathological appearance with organ site specificity. Tumor metastases within the abdominal cavity were pink-white and soft masses (Fig. 2a) with mostly hepatocellular differentiation (Fig. 2c) either well-differentiated with a trabecular pattern or moderately-to-poorly differentiated with no evidence of trabeculae. Within these tumors, focal well-differentiated adenocarcinoma elements highlighted by CK19 staining were also observed, suggesting a cholangiocellular differentiation. Single small cells with scant cytoplasm and hyperchromatic, oval nuclei highlighted by intensive cytoplasmic CK19 staining were present within these tumors, possibly representing transformed progenitors. CK19-positive neoplastic cells with an increased nucleus:cytoplasm ratio and marked pleomorphism were also observed in regions with hepatocellular differentiation. By contrast, the subcutaneous metastasis on the back was a grey-white, firm and solid mass with CC predominance histologically highlighted by CK19 staining (Fig. 2a, d). Broader desmoplastic stroma was observed at the subcutaneous metastasis margin than in intrahepatic tumor. In the splenic metastasis (Fig. 2e), areas of moderately-poorly differentiated HCC mingled with well-moderately differentiated CC, surrounded by areas of well-differentiated HCC with mild CK19 staining, suggesting a multistep hepatocarcinogenesis or multicentric occurrence.

Collectively, these data suggest that the heterogeneity of the CHC metastases tumors may result from multistep maturation from progenitors to tumor cells by spontaneous differentiation or diverse local environmental tropism and choices.

### Intermediate areas of murine tumors mimic that of humans: histological correspondence and variance according to WHO criteria

The intermediate area of CHC with stem cell features has drawn significant attention in this field due to its unique histological appearance. As WHO criteria suggest, intermediate areas of human CHCs with stem cell features can be classified into 3 subtypes: typical subtype, intermediate-cell subtype, and cholangiolocellular subtype [22]. Macroscopic and microscopic pathological profiles of human resected CHCs are summarized in Table 1. Immunohistochemical profiles are summarized in Table 2 to supplement those previously described [23]. We attempted to define corresponding elements within murine hepatoblast-derived liver tumor accordingly by presenting pathological images from human and murine tumors in Fig. 3, as follows.

**Table 2:** Immunohistochemical profiles of 8 patients diagnosed as combined hepatocellular-cholangiocarcinoma with stem cell feature

	HepPar1	CK7	CK19	AFP	OV-6	EpCAM	c-kit	CD133
CHC with stem cell features (n = 8)	5	8	7	7	8	8	8	5

*AFP* alpha-fetoprotein, *EpCAM* epithelial cell adhesion molecule, *CK* cytokeratin, *CHC* combined hepatocellular-cholangiocarcinoma

### CHC with stem cell features, typical subtype

Typical subtype mimics a maturation process from the peripheral undifferentiated HPC to central mature hepatocytes within regenerative nodules (Fig. 3a, b). In murine samples (Fig. 3a), intermediate areas of the tumor contains peripheral clusters of small oval-shaped cells at the margins of nests of HCC, compared to thin strands of parallel small cells with scanty cytoplasm and hyperchromatic nuclei enclosing nests of neoplastic hepatocytes with clear cytoplasm in human tumor section (Fig. 3b). Similar to CC-like areas, minor formation of fibrous stromal in the murine tumor is observed, in contrast with dramatic desmoplastic stromal formation within human tumors.

### CHC with stem cell features, intermediate-cell subtype

Intermediate-cell subtype is characterized by small neoplastic cells with features intermediate between hepatocytes and cholangiocytes (Fig. 3c, d). In human CHC (Fig. 3d), the Oval-shaped HPC-like cells were observed, arranged in solid nests or strands and embedded in a background of marked desmoplasia. A corresponding histological appearance could also be identified in murine tumors (Fig. 3c) [24].

### CHC with stem cell features, cholangiolocellular subtype (foci)

**Fig. 4:**
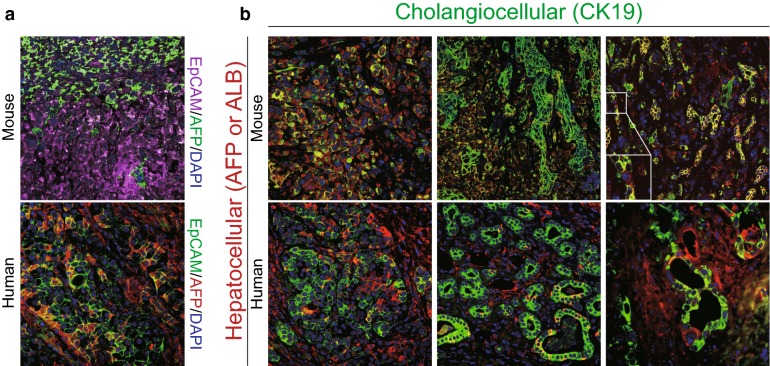
Both murine p53^−/−^ hepatoblast-derived tumors and human cholangiohepatoma exhibit similar differentiation patterns. **a** Fields of double-immunofluorescence staining for EpCAM/AFP murine and human tumors are shown. In murine tumors (upper image), cells showing membrane and cytoplasmic EpCAM staining (purple) co-localizing with cytoplasmic AFP (green) are shown. In human tumors (lower image), similar cells showing weak cytoplasmic EpCAM staining (green) co-localizing with cytoplasmic AFP (red) appear to be orange in cytoplasm and green in membrane. **b** Images of double-immunofluorescence staining for CK19/ALB in murine tumors and CK19/AFP in human tumors are presented. Representative fields of intermediated area (left), cholangiocellular carcinoma (middle), and cholangiolocellular carcinoma (right) are shown. Tumor cells with intermediate phenotype appear to be yellow/orange

Cholangiolocellular subtype, which is known formerly as cholangiolocellular carcinoma (CLC), is thought to originate from the ductules and/or canals of Hering due to its similarity with ductular reactions (DR) (Fig. 3e, f) [6]. Cholangiolocellular components have small tumor cells with high nuclear:cytoplasmic ratio and hyperchromatic, oval nuclei. The tumor cells tend to grow in a monotonous tubular, cord-like, anastomosing pattern embedded in a fibrous stroma, also known as an “antler-like” pattern. Cholangiolocellular components appearing to recapitulate DR could be occasionally observed in both murine (Fig. 3e) and human tumors (Fig. 3f). In addition to the above-mentioned hepatocellular maturation-like appearance denoting mature-appearing hepatocytes in contiguity with malignant ductules (Figs. 2e, 4b), CC components are also found contiguous with cholangiolocellular components in our murine model, further supporting the bidirectional differentiation potential of the transformed hepatoblasts.

### Phenotypic correspondence of murine and human tumors

We have reported and also presented in Fig. 4 that intermediate foci with HPC-like phenotype were common in human CHCs, identified by co-expression of HPC/biliary markers (e.g., CK19 or EpCAM), and immature hepatocyte markers (e.g., AFP) [1]. By multicolor fluorescence immunostaining, we confirmed that the murine tumor cells within intermediate area shared similar intermediate phenotype with cultured hepatoblasts which simultaneously express biliary marker (e.g., CK19), hepatocellular marker (e.g. ALB). However, we only occasionally observed AFP^+^ tumor cells in murine tumors. Furthermore, the few AFP^+^ tumor cells are always found to be negative for EpCAM staining confirmed by double immunofluorescence (Fig. 4a). Thus, it might not be possible to utilize AFP as a hepatocellular marker in murine immunofluorescence assays unlike in the human samples. Not only in intermediate areas, but also in CC or CLC areas of murine tumor, tumor cells with intermediate phenotype featured by co-expressing CK19 and ALB were found. In CLC area, above-mentioned ALB^+^CK19^−^ mature-appearing hepatocytes were contiguous with ALB^+^CK19^+^ and ALB^−^CK19^+^ malignant ductules, similar to ductular reactions (DR) with hepatocyte regeneration (Fig. 4b). Collectively, though with limited markers, we consider that the intermediate phenotype in both human CHC and murine hepatoblast-derived tumors may be associated with a HPC trait. The development of CHC may recapitulate HPC initiated liver regeneration.

## Discussion

Combined hepatocellular-cholangiocarcinoma is a subtype of hepatic tumors with marked heterogeneity suspected to represent a hepatic ‘stem cell’ malignancy [8]. In the most recent edition of WHO diagnostic criteria for CHC, predominance of “stem-cell features” is considered a hallmark of CHC for subclassification [22] and each subtype is associated with variant clinical and pathological significances [25]. Furthermore, cholangiolocarcinoma, traditionally classified as subtype of cholangiocarcinoma, has been reclassified as a subtype of CHC due to expression of HPC-associated signatures [6]. As committed precursors, HPCs can differentiate into either hepatocytes or cholangiocytes [13, 26]. During differentiation, HPCs give rise to malignancy with a spectrum of phenotypes with varying hepatocellular and cholangiocellular features [5]. CHC is thought to originate from HPC via “maturational arrest,” [27] during the process of tumor differentiation.

Accumulating evidence from human tissues support the HPC origin of CHC, by identifying HPC-associated traits in tumor architecture; however, the observational nature of the pathological studies precludes drawing hard conclusions regarding cell origin of CHC since histological and biomarker profiles do not necessarily predict the cell of origin [28]. Most experimental animal studies only demonstrate the involvement of HPC in tumorigenesis but phenotypic characterization of the generated tumors is very limited [13, 15, 16]. Recently, Holczbauer et al. [10] has demonstrated that sequential differentiation stages of hepatic lineage including hepatoblasts, HPCs, and mature hepatocytes can all give rise to liver carcinomas harboring varied proportions of CC, anaplastic tumors, and HCC. In the present study, we compared the pathological characteristics of 8 cases post-operatively diagnosed as CHC with stem cell features and murine E-Cadherin^+^ p53^−/−^ hepatoblast-derived liver tumors. We found that these transformed murine hepatoblasts can generate mixed liver carcinomas greatly mimicking CHC with stem cell features (defined by latest WHO criteria). Specifically, every described subtype of human CHCs matched with corresponding areas in murine tumors. In both murine and human tumors, small, hyperchromatic, ‘oval-like’ cells in peripherally located tumor nests with stem-like features are suggested histologically to be the progenitor origin of the cancer, since the nests of cells appear to be at the head of a progressive maturation process from the undifferentiated small cells at the periphery to the mature cells at the center [8]. These areas of partial differentiation strongly resemble ductular reactions at the interface of the portal and parenchymal compartments during regeneration. In our murine model, transplanted progenitors generated tumor nodules that appear to manifest a process of maturation from progenitors to differentiated tumor cells. However, it remains impossible to rule exclude that the observed “stemness” or CC elements in the murine CHC could be the result of de-differentiation or trans-differentiation of HCCs since recent data suggest that a simultaneous activation of NOTCH and AKT signaling in hepatocytes can trigger cholangiocellular transdifferentiation and the growth of CC [29–31].

Genetic lineage tracing mediated by Cre recombinase has opened up the door for scientists to explore the cellular events in hepatocarcinogenesis. Numerous mouse models of hepatocarcinogenesis have demonstrated mature hepatocytes primarily generate HCC through malignant transformation under selective pressure induced by chronic inflammatory milieu [12, 32, 33]. An elegant cholangiocyte-lineage tracing study also has addressed the contribution of mature cholangiocyte to CC development [34]. Earlier lineage tracing studies in commonly used murine hepatocarcinogenesis models precluded finding HPC to be the origin of PLC [35]. Recently, by applying genetic lineage tracing in a novel mouse model mimicking human multistep liver tumor development, Tummala et al. [11] demonstrate that both mature hepatocytes and HPCs participate in hepatocarcinogenesis and subsequence liver tumor heterogeneity. The variability of results may due to various murine models with differential capacity of mimicking the human disease. Thus, the cellular origin of PLC including CHC remains complex warranting further investigation.

It is noteworthy that formation of fibrous stroma within murine orthotopic tumor was modest in our model. In human CC, the presence of a dense desmoplastic stroma is a prominent phenotype [36]. The stroma and its mesenchymal cell components, particularly cancer-associated fibroblasts (CAFs), have been recognized to play a crucial role in liver tumor progression in recent years. The cellular origin and molecular mechanisms of CAF recruitment or induction have not been fully elucidated yet. CAFs are thought to be derived from hepatic stellate cells, portal fibroblasts, bone marrow-derived precursor cells, or hepatoma cells through an epithelial–mesenchymal transition (EMT) process [37]. In our protocol, we did not administer a fibrogenic dose of CCl_4_, potentially limiting the genesis of CAFs in our model. It is also possible that the inflammatory milieu created by CCl_4_ administration cannot fully mimic human chronic liver disease, since liver tumor phenotype has been suggested defined by nature of chronic liver inflammation. Broad desmoplastic stroma found in our margin area of subcutaneous metastasis may support the idea. However, detailed mechanism will be interesting and should be investigated in the future.

## Conclusion

The pathological characteristics of murine hepatoblast-derived tumors recapitulate human CHC with stem cell features. The current study provides additional comparative pathological evidence that CHC with stem cell features originate from a progenitor cell. It is not surprising that causal relationship still cannot be demonstrated unless a convincing endogenous biomarker is innovated for in vivo tracking of HPC. Further study into the mechanisms by which HPC participate hepatocarcinogenesis, including epigenetic or somatic changes of tumor initiating cells during malignant transformation and interactions between microenvironment and tumor-initiating cells, will clarify the causal relationship between HPC and hepatocarcinogenesis.

## Supplementary information

**Additional file 1: Supporting Tables.** List of antibodies and primers.

**Additional file 2.** Supporting materials and methods.

## Data Availability

The datasets generated during and/or analyzed during the current study are available from the corresponding author on reasonable request.

## Abbreviations

CHC: Combined hepatocellular-cholangiocarcinoma
HCC: Hepatocellular carcinoma
CC: Cholangiocarcinoma
DR: Ductular reaction
AFP: α-Fetoprotein
CK7: Cytokeratin 7
CK19: Cytokeratin 19
IHBC: Intermediate hepatobiliary cell
HPC: Hepatic progenitor cell
PLC: Primary liver cancer
CLC: Cholangiolocellular carcinoma
WHO: World Health Organization
GFP: Green fluorescent protein
