# Supplementary material for: Murine hepatoblast-derived liver tumors resembling human combined hepatocellular-cholangiocarcinoma with stem cell features
Source: Cell Biosci. 2020 Mar 13;10:38. doi: 10.1186/s13578-020-00395-2 (PMC7071781; doi:10.1186/s13578-020-00395-2)
Supplement: Supplementary file 1 — Additional file 1: Supporting Tables. List of antibodies and primers. [file 13578_2020_395_MOESM1_ESM.docx]

| **Table S1. List of Antibodies Used** | | | | | |
| --- | --- | --- | --- | --- | --- |
| **Primary antibody** | **Clone** | **Species** | **Reference** | **Dilution** | **Description** |
| CK19 | RCK 108 | Mouse MAb | Dako, Glostrup, Denmark | 1: 50 | cholangiocyte |
|  | EPNCIR127B | Rabbit MAb | Epitomics, Burlingame, CA | 1: 250 |  |
| AFP | EP1017Y | Rabbit MAb | Abcam, Cambridge, MA | 1:250 | Immature hepatocyte |
|  |  | Rabbit PAb | Dako | 1: 500 |  |
| EpCAM | Ber-EP4 | Mouse MAb | Dako | 1:400 | Oval cells/Progenitors |
|  | G8.8 | Rat MAb | eBioscience, San Diego, CA | 1:100 |  |
| E-Cadherin | 114420 | Rat MAb | R&D, Minneapolis, MN | 1:100 | Epithelium |
|  | ECCD-1 | Rat MAb | Calbiochem, San Diego, CA | 1:100 |  |
| ALB |  | Sheep PAb | Abcam | 1:1000 | hepatocyte |
|  |  | Rabbit PAb | AbD Serotec, Raleigh, NC | 1:200 |  |
| CD133 | 13A4 | Rat MAb | eBioscience | 1:100 | Oval cells/Progenitors |
| GFP |  | Rabbit PAb | Abcam | 1:1000 | fluorescent protein |
| CK7 | OV-TL 12/30 | Mouse MAb | Dako, Glostrup, Denmark | 1: 50 | cholangiocyte |
| HepPar1 | OCH1E5 | Mouse MAb | Dako | 1:100 | Human Hepatocyte |
| OV-6 | OV-6 | Mouse MAb | R&D, Minneapolis, MN | 10μg/mL | Oval cells/Progenitors |
| c-Kit |  | Rabbit PAb | Dako | 1:400 | Stem/Progenitor cells |
| CD133 |  | Rabbit PAb | Novus Biologicals, Littleton, CO | 1:100 | Oval cells/Progenitors |
| Abbreviations: AFP, alpha-fetoprotein; ALB, albumin; EpCAM, epithelial cell adhesion molecule; GFP, green fluorescent protein; MAb, Monoclonal antibody; PAb, polyclonal antibody. | | | | | |

| **Table S2. Primers used in RT-PCR** | | |
| --- | --- | --- |
| **Gene name** |  | **Sequences** |
| DLK1 | Sense | 5’-GTGCGAAACCTGGGTGTCC-3’ |
|  | Antisense | 5’-GCCTCCTTGTTGAAAGTGGTCA-3’ |
| SOX9 | Sense | 5’-CAGCCCCTTCAACCTTCCTC-3’ |
|  | Antisense | 5’-TGATGGTCAGCGTAGTCGTATTG-3’ |
| EPCAM | Sense | 5’-GCGGCTCAGAGAGACTGTGTC-3’ |
|  | Antisense | 5’-CCAAGCATTTAGACGCCAGTTT-3’ |
| G6PC | Sense | 5’-CGACTCGCTATCTCCAAGTGA-3’ |
|  | Antisense | 5’-GTTGAACCAGTCTCCGACCAC-3’ |
| KRT7 | Sense | 5’-TTGCTGAAGAAGGATGTGGATG-3’ |
|  | Antisense | 5’-TCTGCTAACTCTGTCTCGTGAAGG-3’ |
| KRT19 | Sense | 5’-GGGTTCAGTACGCATTGGGT-3’ |
|  | Antisense | 5’-CGGAGGACGAGGTCACGA-3’ |
| MYC | Sense | 5’-TGGATTTCCTTTGGGCGTT-3’ |
|  | Antisense | 5’-CTCGCTCTGCTGTTGCTGG-3’ |
| NANOG | Sense | 5’-TCTTCCTGGTCCCCACAGTTT-3’ |
|  | Antisense | 5’-GCAAGAATAGTTCTCGGGATGAA-3’ |
| TDO2 | Sense | 5’-ATGAGTGGGTGCCCGTTTG-3’ |
|  | Antisense | 5’-GGCTCTGTTTACACCAGTTTGAG-3’ |
| AAT1 | Sense | 5’-CTAGGGAGCAAGGGTGACACTC-3’ |
|  | Antisense | 5’-CACTGTCTGGTCTGTTGAGGGTT-3’ |
| AFP | Sense | 5’-GCTTCCCTCATCCTCCTGCTAC-3’ |
|  | Antisense | 5’-AACAAACTGGGTAAAGGTGATGG-3’ |
| ALB | Sense | 5’- GGTGTGTTTCGCCGAGAAGCAC -3’ |
|  | Antisense | 5’- GGCGGCAGACTCATCGGC -3’ |
| GAPDH | Sense | 5’-CCGTGTTCCTACCCCCAAT-3’ |
|  | Antisense | 5’-ATGCCTGCTTCACCACCTTC-3’ |
