# Supplementary material for: Murine hepatoblast-derived liver tumors resembling human combined hepatocellular-cholangiocarcinoma with stem cell features
Source: Cell Biosci. 2020 Mar 13;10:38. doi: 10.1186/s13578-020-00395-2 (PMC7071781; doi:10.1186/s13578-020-00395-2)
Supplement: Supplementary file 2 — Additional file 2. Supporting materials and methods. [file 13578_2020_395_MOESM2_ESM.doc]

***Additional methods***

***Generation of p53^-/-^ hepatoblasts***

B6.129S2-*Trp53^tm1Tyj^*/J (p53^+/–^) mice were purchased from Jackson Lab (Rochester, New York), and were housed in a specific pathogen free animal facility. Female p53^+/–^ mice were crossed with male p53^–/–^ mice to generate p53^–/–^ and p53^+/–^ offspring. Ultimately, p53^–/–^ male mice and p53^–/–^ female mice were bred yielding p53^–/–^ embryos harvested at E13.5. Purification of E-Cadherin+ hepatoblasts from embryonic liver suspensions was performed using the MACS® magnetic cell-sorting system (Miltenyi, Auburn, California) with the rat anti-mouse E-Cadherin (ECCD-1) antibody (Calbiochem, San Diego, California) and goat anti-rat IgG microbeads. Purification was performed by the protocol recommended by the manufacturer via indirect labeling. The positive selection of E-Cadherin+ cells was plated in a 6-well plate pre-coated with Matrigel matrix (Corning, Corning, New York). The purity of sorted cells was accessed by flow cytometry using PE-conjugated rat anti-mouse E-Cadherin (114420) antibody (R&D Systems, Minneapolis, Minnesota). Purified hepatoblasts were plated on Mitomycin-C pre-treated STO feeder cells in a hepatocyte growth medium supplemented with hepatocyte growth factor (HGF) (20 ng/ml) (Peprotech, Rocky Hill, New Jersey) and epidermal growth factor (EGF) (20 ng/ml) (Peprotech, Rocky Hill, New Jersey) to maintain an undifferentiated state during long-term culture.[1] After 2 passages, a subset of cultured hepatoblasts were transduced with pMYs-IRES-EGFP retroviral vector as described previously. In brief, retrovirus was produced in Platinum-E packaging cells and collected in hepatocyte growth medium. Supernatant was passed through a 0.45µm filter and supplemented with polybrene (5µg/ml). Transfection was performed for 6 hours. 48 hours after transfection, the fraction of EGFP+ cells was assessed by fluorescence microscopy and flow cytometry.

***Surgical procedures***

Mice under general anesthesia with isoflurane underwent an epigastric incision. After splenic vascular pedicle exposure, the upper and lower pole of the spleen were separated by two suture loops created by square knot between the upper and lower branch of the vasculature. 1 × 10^6^ hepatoblasts were then injected into the lower pole of the spleen. Lower branch of the vascular pedicle was ligated and the lower pole of the spleen was removed. The abdominal wall was then closed with 3-0 suture.

***Flow cytometry and cell sorting***

Staining of cultured hepatoblasts was performed on single-cell suspensions as described. For GFP detection, single-cell suspensions were directly analyzed using FACS Canto flow cytometer (BD Biosciences, San Jose, California). For immunophenotyping of cell surface antigens, fluorescent-labeled antibodies listed in Supporting Table 1 were used for flow cytometry staining. Data were analyzed using FlowJo software (FlowJo, Ashland, OR). To separate GFP+ hepatoblasts, the retrovirus transfected hepatoblasts were sorted with FACS Aria (BD Biosciences, San Jose CA) after excluding debris, doublets, and dead cells by forward scatter, side scatter gating.

***Reverse-Transcription Polymerase Chain Reaction Analysis***

Total RNA was prepared using TRIzol (Takara, Mountain View, California) reagent and was reverse-transcribed using a SMARTer PCR cDNA Synthesis Kit (Takara). Complementary DNA was amplified by polymerase chain reaction (PCR) with the Taq DNA Polymerase (Takara). PCR was performed by using primers listed in Supporting Table 2.

***Tissue processing, histological staining, Immunohistochemistry, and Immunofluorescence***

Dissection, fixation, embedding and storage of specimens were performed based on previously described protocols. [2] The hematoxylin/eosin (H&E) staining and Periodic Acid-Schiff (PAS) staining protocols have been previously described.[3] PAS staining is used for the detection of mucus production assisting diagnosis of CC component in murine tumor. Cultured cells were placed on chambered slides for processing. After fixation with 4% paraformaldehyde, permeabilization with 0.2% Triton, and blocking in 5% bovine serum albumin (BSA), cells were incubated with a primary antibody overnight at 4°C. The cells then were washed and incubated with horseradish peroxidase (HRP)-conjugated or Alexa Fluor® Dyes-conjugated secondary antibodies for 2 hours at room temperature. For immunocytochemistry, the cells were reacted with Dako liquid DAB+ substrate chromogen and counter-stained with hematoxylin following protocol recommended by the manufacturer. For immunofluorescence, the cells were mounted with DAPI containing mounting medium for fluorescence microscope observation.

Tissue slices (4 μm) were sectioned from paraffin blocks, subsequently deparaffinized in xylene and rehydrated through graded alcohols. Endogenous peroxidases were inactivated using 3% H_2_O_2_ in methanol for 20 min. Optimal retrieval conditions depended on the type of tissue, fixation, and antibody used. Antibody incubation and subsequent procedure is same to protocol for cultured cells. To reduce autofluorescence, tissue sections were treated with Sudan black B as described. [4]

***Diagnostic details of combined hepatocellular cholangiocarcinoma***

According to the criteria, the diagnosis of CHC requires unequivocal histologic presence of both hepatocellular and cholangiocellular areas. Intermediate foci with intimately mixed characteristics of both HCC and CC also should be sought to distinguish separate/collision type, but not necessarily located at the interface of the HCC and CC components.[5]

Briefly, typical hepatocellular characteristics were confirmed by trabecular, adenoid or solid pattern of growth and bile production. The cholangiocellular characteristics were identified as small glandular formations composed of small cuboidal cells with round nuclei and no nucleoli resembling a biliary epithelium with abundant fibrous stromal. The intermediate areas were defined as areas in which HCC elements and CC elements were difficult to discriminate. Detailed diagnostic criteria and representative images have been described in our previous publication.[6, 7]

**REFERENCES**

1. Zender L, Xue W, Cordon-Cardo C, Hannon GJ, Lucito R, Powers S, Flemming P, Spector MS, Lowe SW: **Generation and analysis of genetically defined liver carcinomas derived from bipotential liver progenitors**. *Cold Spring Harbor symposia on quantitative biology* 2005, **70**:251-261.

2. Hruban RH, Askin FB: **Surgical pathology dissection : an illustrated guide**. New York: Springer; 1996.

3. Carson F, Hladik C: **Histotechnology: A Self-Instructional Text**, 3rd edn. Chicago, IL: American Society for Clinical Pathology Press; 2009.

4. Viegas MS, Martins TC, Seco F, do Carmo A: **An improved and cost-effective methodology for the reduction of autofluorescence in direct immunofluorescence studies on formalin-fixed paraffin-embedded tissues**. *European journal of histochemistry : EJH* 2007, **51**(1):59-66.

5. Bosman FT, World Health Organization., International Agency for Research on Cancer.: **WHO classification of tumours of the digestive system**, 4th edn. Lyon: International Agency for Research on Cancer; 2010.

6. Cai X, Xiong J, Hu QG, Zhao QD, Wu D, Tang LG, Wan CD, Wei LX: **Look into hepatic progenitor cell associated trait: Histological heterogeneity of hepatitis B-related combined hepatocellular-cholangiocarcinoma**. *J Huazhong Univ Sci Technolog Med Sci* 2017, **37**(6):873-879.

7. Cai X, Zhai J, Kaplan DE, Zhang Y, Zhou L, Chen X, Qian G, Zhao Q, Li Y, Gao L *et al*: **Background progenitor activation is associated with recurrence after hepatectomy of combined hepatocellular-cholangiocarcinoma**. *Hepatology* 2012, **56**(5):1804-1816.
